# Neurohypophysial Hormones Regulate Amphibious Behaviour in the Mudskipper Goby

**DOI:** 10.1371/journal.pone.0134605

**Published:** 2015-07-31

**Authors:** Tatsuya Sakamoto, Yudai Nishiyama, Aoi Ikeda, Hideya Takahashi, Susumu Hyodo, Nao Kagawa, Hirotaka Sakamoto

**Affiliations:** 1 Ushimado Marine Institute, Faculty of Science, Okayama University, Ushimado, Setouchi, 701–4303, Japan; 2 Laboratory of Physiology, Atmosphere and Ocean Research Institute, University of Tokyo, 5-1-5 Kashiwanoha, Kashiwa, Chiba, 277–8564, Japan; 3 Department of Life Science, Faculty of Science and Technology, Kinki University, Higashiosaka, Osaka, 577–8502, Japan; University of Macau, MACAO

## Abstract

The neurohypophysial hormones, arginine vasotocin and isotocin, regulate both hydromineral balance and social behaviors in fish. In the amphibious mudskipper, *Periophthalmus modestus*, we previously found arginine-vasotocin-specific regulation of aggressive behavior, including migration of the submissive subordinate into water. This migration also implies the need for adaptation to dehydration. Here, we examined the effects of arginine vasotocin and isotocin administration on the amphibious behavior of individual mudskippers *in vivo*. The mudskippers remained in the water for an increased period of time after 1–8 h of intracerebroventricular (ICV) injection with 500 pg/g arginine vasotocin or isotocin. The ‘frequency of migration’ was decreased after ICV injection of arginine vasotocin or isotocin, reflecting a tendency to remain in the water. ICV injections of isotocin receptor antagonist with arginine vasotocin or isotocin inhibited all of these hormonal effects. In animals kept out of water, mRNA expression of brain arginine vasotocin and isotocin precursors increased 3- and 1.5-fold, respectively. Given the relatively wide distribution of arginine vasotocin fibres throughout the mudskipper brain, induction of arginine vasotocin and isotocin under terrestrial conditions may be involved also in the preference for an aquatic habitat as ligands for brain isotocin receptors.

## Introduction

In mammals, the neurohypophysial hormones, arginine-vasopressin (VP) and oxytocin (OXY), are produced by neurones localised throughout the hypothalamic regions. These neurones project into the pituitary and into multiple extrahypothalamic regions in vertebrates, and are known to play pivotal roles in osmoregulation, stress response, reproduction, and mediation of social behaviours [1,2,3,4,5,6,7]. In teleost fishes, an association of arginine-vasotocin (VT), the teleost homologue of mammalian VP, with regulation of hydromineral balance and social behaviour has been shown [8,9,10,11,12,13,14,15,16,17,18]. However, the possible link between osmoregulation and aggressive behavior, both of which are controlled by the same peptide, has received relatively little attention, even in mammals.

Mudskipper fishes are amphibious and spend the greater part of their lives out of water to feed and to avoid capture by aquatic predators. Therefore, they have behavioural and physiological specializations adapted to amphibious life [19,20,21,22,23]. In the mudskipper, *Periophthalmus modestus*, we have recently shown VT-specific regulation of general aggressive behaviour and/or social communication (fin display, operculum display, replacement, attacking, chasing, biting) and a characteristic role of VT in promoting a longer length of stay in the water for submissive subordinates relative to aggressive dominants. This migratory behavior into water cannot be fully explained by a physiological stress response, since no difference in plasma cortisol levels was detected [24]. Given the suite of processes mediated by neurohypophysial hormones, migration of subordinate mudskippers into water may reflect a unique interaction between the hormonal regulation of social behaviours and adaptation from dehydrating conditions on land to aquatic environments in amphibious fishes. Thus, this mudskipper is a valuable experimental model to provide new insights into the role of VT, and the direct involvement of neurohypophysial hormones in amphibious behaviour is highly intriguing. Here, we performed *in vivo* administration of VT and isotocin (IT, the teleost homologue of OXY), and found that these hormones mediated the preference for an aquatic habitat in the individual mudskipper. We also analyzed pro-VT and pro-IT expression in the brain during terrestrial adaptation by real-time polymerase chain reaction (PCR) assays for mudskipper VT and IT precursor mRNA. We also investigated the organisation of VT and IT neurones to suggest the region in which these hormones exert effects in the brain.

## Materials and Methods

### Animals

This study was approved by the Committee for Animal Research, Okayama University (IACUC), and conforms to the Guidelines for Animal Experimentation established by Okayama University. No specific permissions were required for the field locations and activities, and the field study did not involve endangered or protected species. The animals were anesthetized before being handled and all efforts were made to minimize suffering.

Four-hundred adult mudskippers (*P*. *modestus*) weighing 4 to 6 g were collected from the estuary of the Fujii River, which flows into the Inland Sea of Seto (34° N: 134° E). Plasma ions, differentiation of osmoregulatory organs, hormonal status, and amphibious behaviours in mudskippers under varying conditions have been described and no sex differences have been found in our previous reports [21,22,23,24,25,26,27,28] or by others [29]. Therefore, both sexes of the fish were used. Fish were acclimated for 2–5 weeks in laboratory tanks (3 L). Since these fish were collected from brackish water, tank water was maintained isotonic with diluted seawater (10 ppt, 149 mM Na, 176 mM Cl−, 3.8 mM Ca, 346 mOsml/kg). All specimens were maintained at a temperature of 22°C to 25°C under a daily photoperiod cycle of 12-h light/12-h dark (lights on at 7:00 a.m.) and were fed daily with Tetrafin flakes (TetraWerke, Melle, Germany). Small plates were placed in each tank to allow mudskippers an opportunity to climb on to them. Before being handled, the fish were anesthetized with 0.01% tricaine methanesulfonate (Sigma, Tokyo, Japan), which was neutralized with sodium bicarbonate. The time to loss of equilibrium after anesthesia was 4–5 min and consistent for each fish. Animals were sacrificed by deep anesthetization.

### Intramuscular injections

The fish were injected intramuscularly with vehicle (0.01% Triton X-305 in saline), vehicle plus VT (Peptide Institute, Osaka, Japan) or plus IT (Biogenesis, Kingston, USA) at doses of 0.1, 1, 10 or 100 ng (0.1, 1, 10 or 100 pmol) /5 μl/g body weight. We chose these doses/treatments based on the results of preliminary studies and published reports on effective physiological doses and plasma hormone concentrations [22,28,30,31,32].

### Intracerebroventricular (ICV) injections

The effects of ICV injections of VT and IT were studied to examine whether these peptides act centrally to affect amphibious behaviour. We previously validated an ICV injection procedure that allowed delivery of vehicle and drugs directly into the third ventricle of several teleost species, including neurohypophysial hormones in this mudskipper species [22,24,33,34]. Anesthetized fish were injected post-orbitally (using a 26-gauge needle), along the midline to a depth of 1 mm into the third ventricle of the brain with one of the drug solutions to achieve a dose of 5 or 500 pg of VT or IT /0.2 μl of artificial cerebrospinal fluid (ACSF) /g body weight. The doses of neurohypophysial hormones were chosen based on the results of preliminary studies and published reports on effective physiological doses [24,35]. Because ICV treatment with VT or IT induced a preference for the aquatic habitat (see Results section) and expression studies of teleost V1a-type receptor (shown to mediate many behaviours [5,36]) and IT receptor in mammalian cell lines indicate that the V1a is almost specific to VT whereas the IT receptor was sensitive to IT and VT [37,38,39,40], involvement of the IT receptor in this amphibious behaviour was suggested. Therefore, we further examined the effects of ICV treatment with 2 ng (2 pmol) [d(CH_2_)_5_^1^, Tyr(Me)^2^, Thr^4^, Orn^8^, Tyr-NH_2_^9^]-VT (H-9405; Bachem, Torrance, CA, USA), or a co-injection of 2 ng H-9405 and 500 pg of VT or IT. Watanabe et al. [35] and Sakihara et al. [41] used this dose of H-9405 to specifically induce IT-receptor blockade. Fish treated in the same way with only ACSF served as handling controls. Evans blue (0.1%) was used to determine the accuracy of the ICV procedure. To minimize the leakage of drugs or ACSF from the injection site, vehicle or drugs were injected slowly, with 5 s allowed to elapse after the injection before the needle was withdrawn.

### Behavioural testing

**Fig 1:**
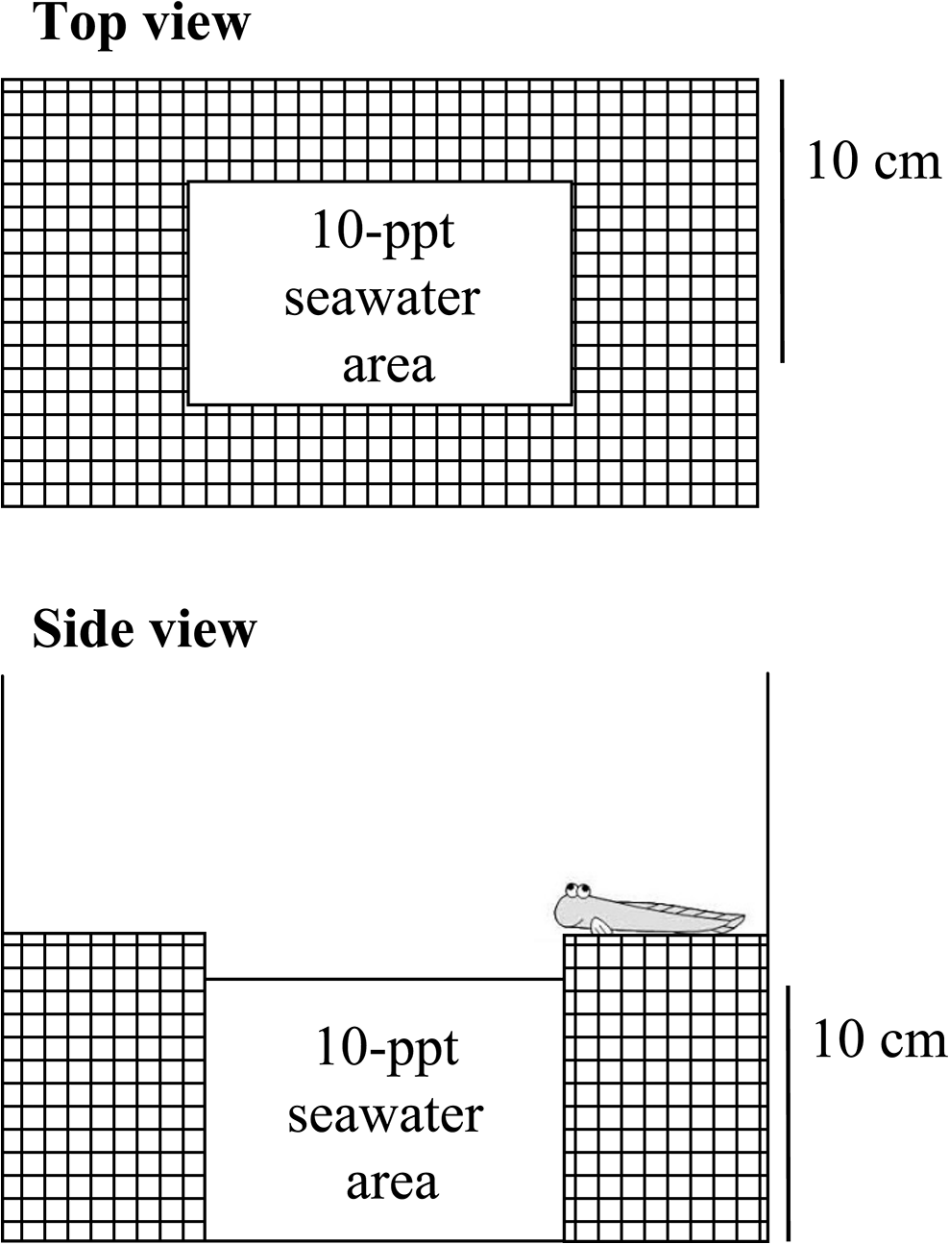
Schematic diagram of the apparatus used to examine the aquatic or terrestrial preference of mudskippers.

Immediately after injection of vehicles and drugs between 09.00 and 10.00 a.m., each fish was placed in the 10-ppt seawater area in an individual aquarium with a land area (Fig 1) [22]. The land area was made of plastic mesh, and care was taken to ensure that there was minimum water on this area. In the water area, aeration was used to ensure oxygen saturation. Full recovery from anesthesia occurred within 1 to 2 min. For each fish, the period of time in the water and the frequency of movement between water and land (defined as the ‘frequency of migration’) were recorded for 8 h.

### Expression of pro-VT and pro-IT mRNAs during terrestrial adaptation

As previously described [21,22,23,27], each intact mudskipper was transferred from 10-ppt seawater and were placed into an individual aquarium containing 10-ppt seawater (control) or an aquarium without water. The control fish could climb on the land area of the aquarium ad libitum. At 5 h, based on studies in mudskippers indicating that many physiological changes occur at this time point following water loss of up to 20% of initial body mass [21,22,23,27,42], the brain, rather than the hypothalamus for dissection reproducibility, was immediately removed, frozen in liquid nitrogen, and then kept at −80°C.

To determine the amount of pro-VT and IT mRNAs by quantitative real-time PCR, partial cDNA sequences of pro-VT and pro-IT were determined for primer design [24]. Total RNA was obtained from the mudskipper brain using an RNeasy Plus Mini kit (Qiagen), which allows efficient removal of genomic DNA. The RNA was quantified using a Qubit Fluorometer (Invitrogen). First-strand cDNA was reverse-transcribed using the SMART RACE cDNA Amplification kit (Clontech). The primer 5′-AAG CAG TGG TAT CAA CGC AGA GTA C(T)30VN-3′ was used for reverse transcription. Degenerate sense primers (5′-GCC TGY TAC ATH TCN AAY TGY CC-3′) designed based on the amino acid sequences of neurohypophysial hormones and anchor primers (5′-CTA ATA CGA CTC ACT ATA GGG CAA GCA GTG GTA TCA ACG CAG AGT-3′ and 5′-CTA ATA CGA CTC ACT ATA GGG C-3′) were used for PCR to clone the 3′ partial region of mudskipper neurohypophysial hormone cDNA. During PCR, the 50 μl reaction mixture (3.5 μl first-strand cDNA, 0.5 μM sense primer, 0.04 μM long anchor primer, 0.2 μM short anchor primer and 25 μl Hot-StarTaq master mix; Qiagen) was subjected to 37 cycles of amplification. After activation of Taq polymerase at 95°C for 15 min, each cycle consisted of a 1 min denaturation at 94°C, 55 s primer annealing at 59°C and 1 min primer extension at 72°C. The final extension was 10 min at 72°C. PCR-amplified cDNA products of expected size (751 bp for VT and 650 bp for IT) were observed after agarose gel electrophoresis (visualized by ethidium bromide staining). DNA was extracted from gels and ligated into pGEM-T Easy Vector (Promega). Nucleotide sequencing of both strands was performed using an ABI 3130xl Genetic Analyzer (PE Applied Biosystems). Multiple clones were examined. These partial sequences were identified as target cDNA fragments because the optimized alignment of the corresponding regions of perciform cDNAs [43] revealed 80% agreement among the respective sequences. The topologies of the phylogenic trees of the aligned sequences were in accordance with the known phylogeny of perciforms (Clustal method) and the sequence did not match significantly with other sequences in the database. The partial nucleotide and derived amino acid sequences of mudskipper pro-VT and IT are available from the GenBank nucleotide sequence database under accession Nos. AB697051 and AB697052, respectively.

For real-time PCR, total RNA (10 ng) samples were isolated as above from brains obtained in the terrestrial adaptation experiment and used for synthesis of first strand cDNA by a reverse transcription reaction using iScript Reverse Transcriptase (Bio-Rad). For the sequences obtained and for mudskipper β-tubulin as a housekeeping gene, the following primers were designed using Primer3Plus for VT, 5′-AGA GAG CTG CGT GCT GGA CT-3′ and 5′-GTG TAT CGG TCG TCT TGT GTC TG-3′; IT, 5′-CTG TTA TGG ACG CCC CTT TG-3′ and 5′-TGG TGT CTG GTC TCG ACG GA-3′; and β-tubulin, 5′-GTC CAT GAA GGA GGT GGA TG-3′ and 5′-TTC TGG CGG CAC ACA CTG-3′. Realtime PCR was carried out using a MiniOpticon Real-Time PCR Detection System (Bio-Rad, Osaka, Japan). The PCR mixture (50 μl) contained 1.0 μl cDNA, 25μl iQ SYBR Green Supermix (Bio-Rad), 200 nM each of forward and reverse primers. Amplification was carried out at 65°C for 1 min, 95°C for 5 min, 39 cycles of 95°C for 11 s and 58°C for 33 s. Dissociation curves produced after the reactions showed a single peak. Dilutions of cDNA from all samples were used to construct a relative standard curve for each primer set, relating the initial template copy number to fluorescence and amplification cycle. For each PCR reaction, a no-template control was introduced as a negative control, in which RNase-free water was added to the reaction instead of the template (cDNA) and no amplification was observed. Real-time PCR data was analyzed with MJ Opticon Monitor software 3.1 (Bio-Rad). Results for pro-VT and IT were normalized using the relatively constant β-tubulin mRNA level.

### Immunofluorescence for VT and IT

Brains, cervical spinal cords, and pituitaries were immediately dissected out from the untreated mudskippers acclimated in laboratory tanks, and immersed in Bouin’s fixative solution [saturated picric acid:10% unbuffered formalin:acetic acid = 15:5:1 (v/v)] overnight at 4°C. Subsequently, tissues were dehydrated and embedded in paraffin wax. Serial sections of each tissue were cut transversely or sagittaly on a microtome at 12-μm thickness. After blocking nonspecific binding components with 1% normal goat serum and 1% bovine serum albumin in phosphate buffered saline (PBS: pH 7.4) containing 0.3% Triton X-100 for 30 min at room temperature, the sections were incubated with primary rabbit antiserum against VT (1:20,000) or IT (1:20,000) overnight at 4°C. The antibodies for VT and IT have been shown to be specific for VT and IT neurones in fish, respectively [9]. Alexa Fluor 488-linked anti-rabbit IgG (Molecular Probes, Eugene, OR) was used at dilution 1:2,000 for 1 h at room temperature. Histology of the brain, spinal cord, and pituitary was studied by blue fluorescent Nissl staining (NeuroTrace 435/455, Molecular Probes). Double-immunofluorescence staining of VT and IT was performed to validate the colocalisation of VT and IT. Sections were first incubated with the VT antibody (1:10,000 dilution) overnight at 4°C after blocking nonspecific binding components, as described above. The VT-immunoreactive products were visualized in red with the conventional immunofluorescent method followed by incubation for 1 h with a solution of Dylight 549-linked Fab fragment goat anti-rabbit IgG (Jackson Laboratory, Bar Harbor, ME, USA) at 1:500 dilution for 1 h at room temperature. After rinsing, the sections were immersed overnight at 4°C in a 1:20,000 dilution of IT antiserum. The second-primary immunoreaction was visualized in green by 1-h incubation with Alexa Fluor 488-linked anti-rabbit IgG at a dilution of 1:2,000. Immunostained sections were viewed by confocal laser scanning microscopy (Fluoview FV1000, Olympus, Tokyo, Japan).

### Statistical analyses

The significance of differences between means for the period of time in water and the ‘frequency of migration’ were analyzed using three-way repeated measures analysis of variance (ANOVA), with time within groups (i.e. after application of treatment), drug treatment among or between groups, and drug concentration within groups as factors. Since there was a significant interaction between treatment (drug and concentration) and time, each time was analyzed separately to identify differences among treatments using Dunnett post-hoc test. The simultaneous effects of neurohypophysial hormone and H-9405 were assessed by two-way ANOVA, followed by Dunnett post-hoc test for comparison between means. All data were checked for normality and equal variances. When assumptions of normality or equal variances were not satisfied, non-parametric Kruskal-Wallis tests were used. mRNA levels of pro-VT and IT were compared by Mann-Whitney U test.

## Results

### Intramuscular injections

**Fig 2:**
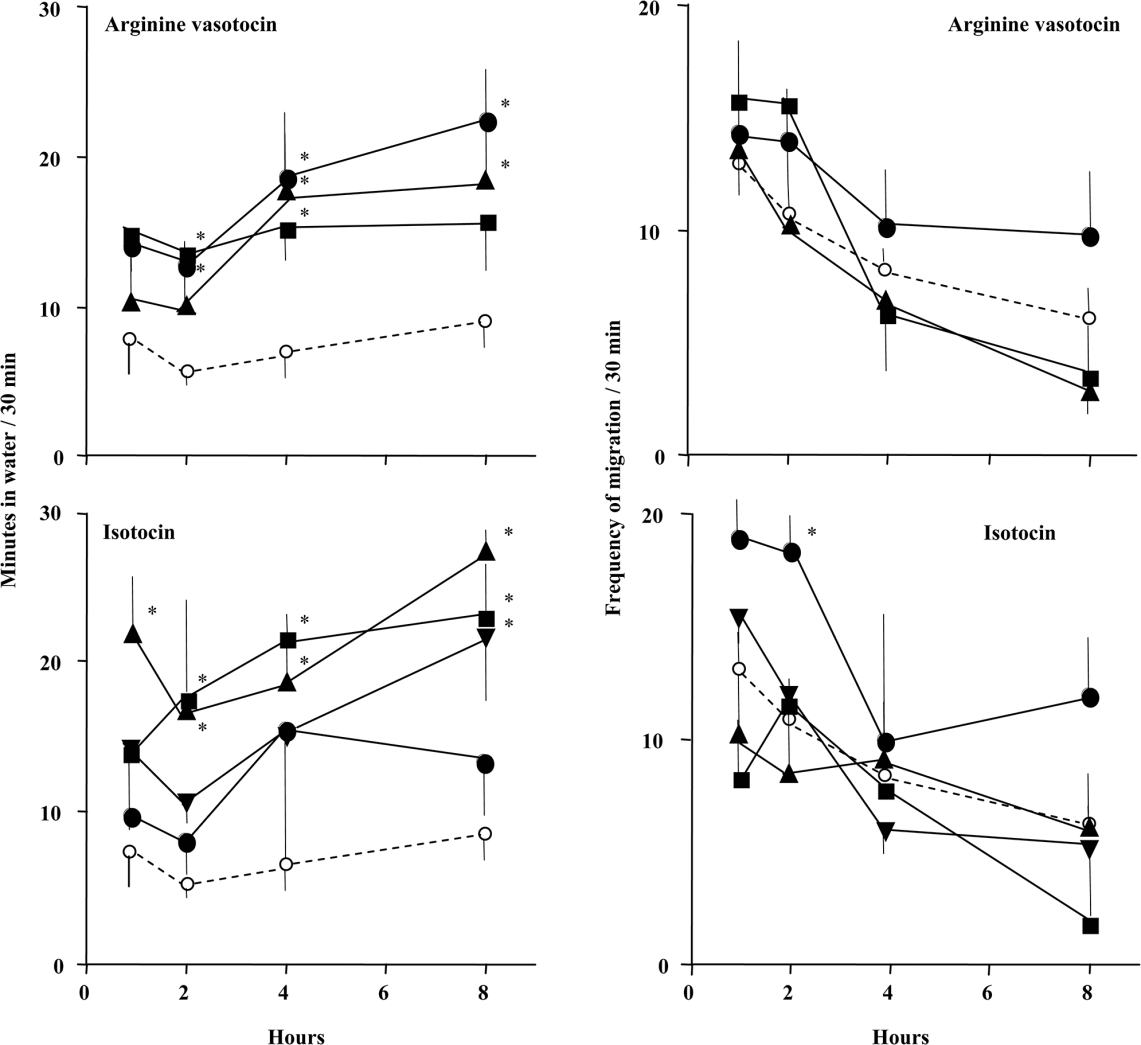
Amphibious behaviour in the mudskipper after intramuscular (i.m.) injection of arginine vasotocin or isotocin. The period of time in water (left panel) and the ‘frequency of migration’ (right panel) for mudskippers after i.m. injection of vehicle (control, open circles), or 0.1 ng/g (inverted triangles), 1 ng/g (triangles), 10 ng/g (squares) or 100 ng/g (solid circles) arginine vasotocin or isotocin. Injection of 0.1 ng/g arginine vasotocin was not performed. The period of time in water and the ‘frequency of migration’ were measured for 30 min at 1 h (0.8–1.3 h), 2 h (1.8–2.3 h), 4 h (3.8–4.3 h) and 8 h (7.8–8.3 h) after treatment. Data are shown as the mean ± standard error (SE) of 4 to 10 fish. The absence of error bars indicates a small SE. *P < 0.05 vs. control at a given time point.

Mudskippers exhibited a significantly increased period of time in water 2–8 h after intramuscular injection of 1–100 ng/g VT and 1–8 h after injection of 0.1–10 ng/g IT, compared with control fish (Fig 2, left panel). There was no clear indication of a significant change in the ‘frequency of migration’ after intramuscular injection (Fig 2, right panel).

### ICV injections

**Fig 3:**
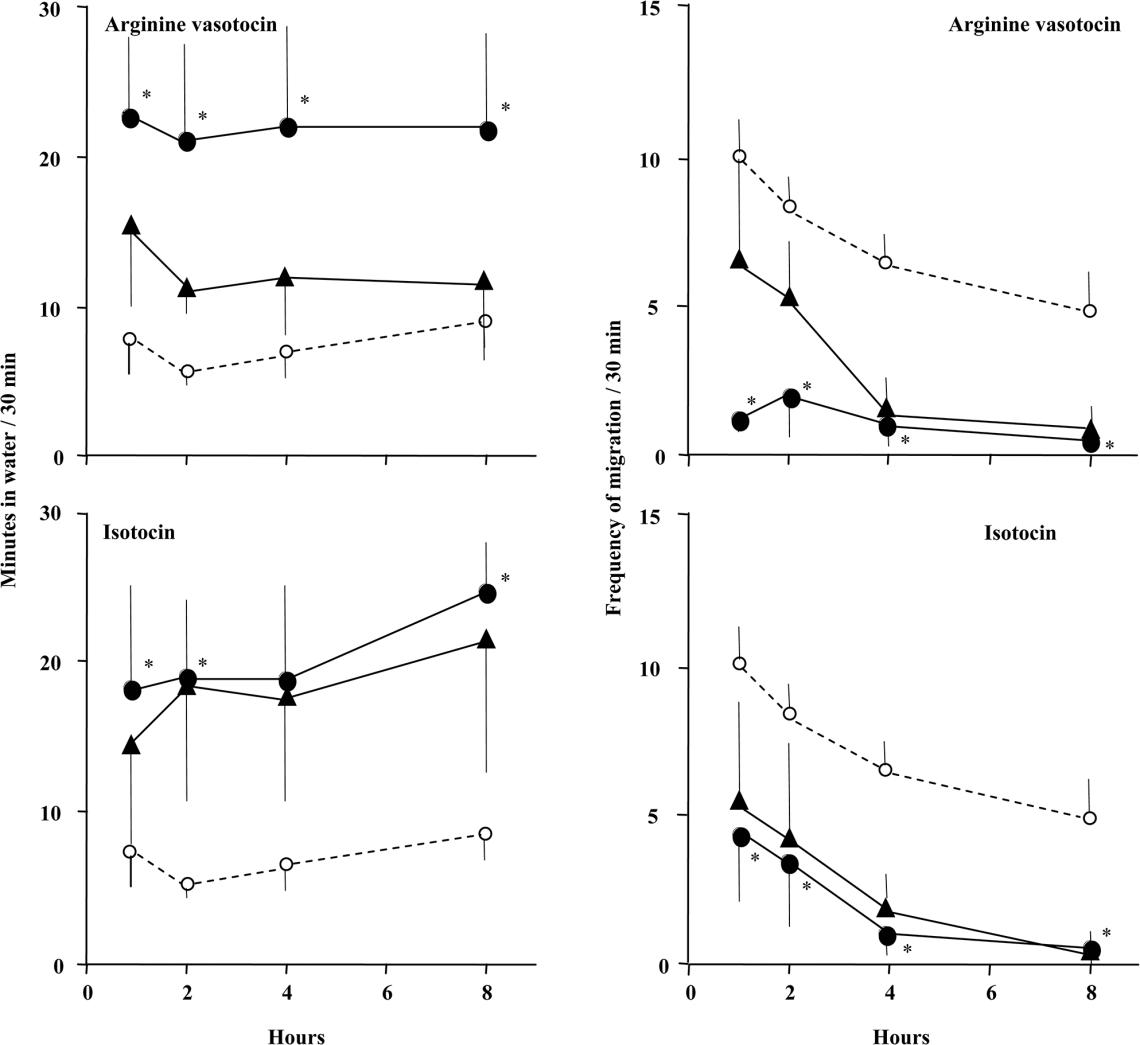
Amphibious behaviour in mudskippers after intracerebroventricular (ICV) injection of arginine vasotocin or isotocin. The period of time in water and the ‘frequency of migration’ for mudskippers after ICV injection of artificial cerebrospinal fluid (control, open circles) or 5 pg/g (triangles) or 500 pg/g (solid circles) arginine vasotocin or isotocin. The period of time in water and the ‘frequency of migration’ were measured for 30 min at 1 h (0.8–1.3 h), 2 h (1.8–2.3 h), 4 h (3.8–4.3 h) and 8 h (7.8–8.3 h) after treatment. Data are shown as the mean ± standard error (SE) of 4 to 11 fish. Only data from ICV procedures whose accuracy was confirmed by Evans blue were used. The absence of error bars indicates a small SE. *P < 0.05 vs. control at a given time point.

**Fig 4:**
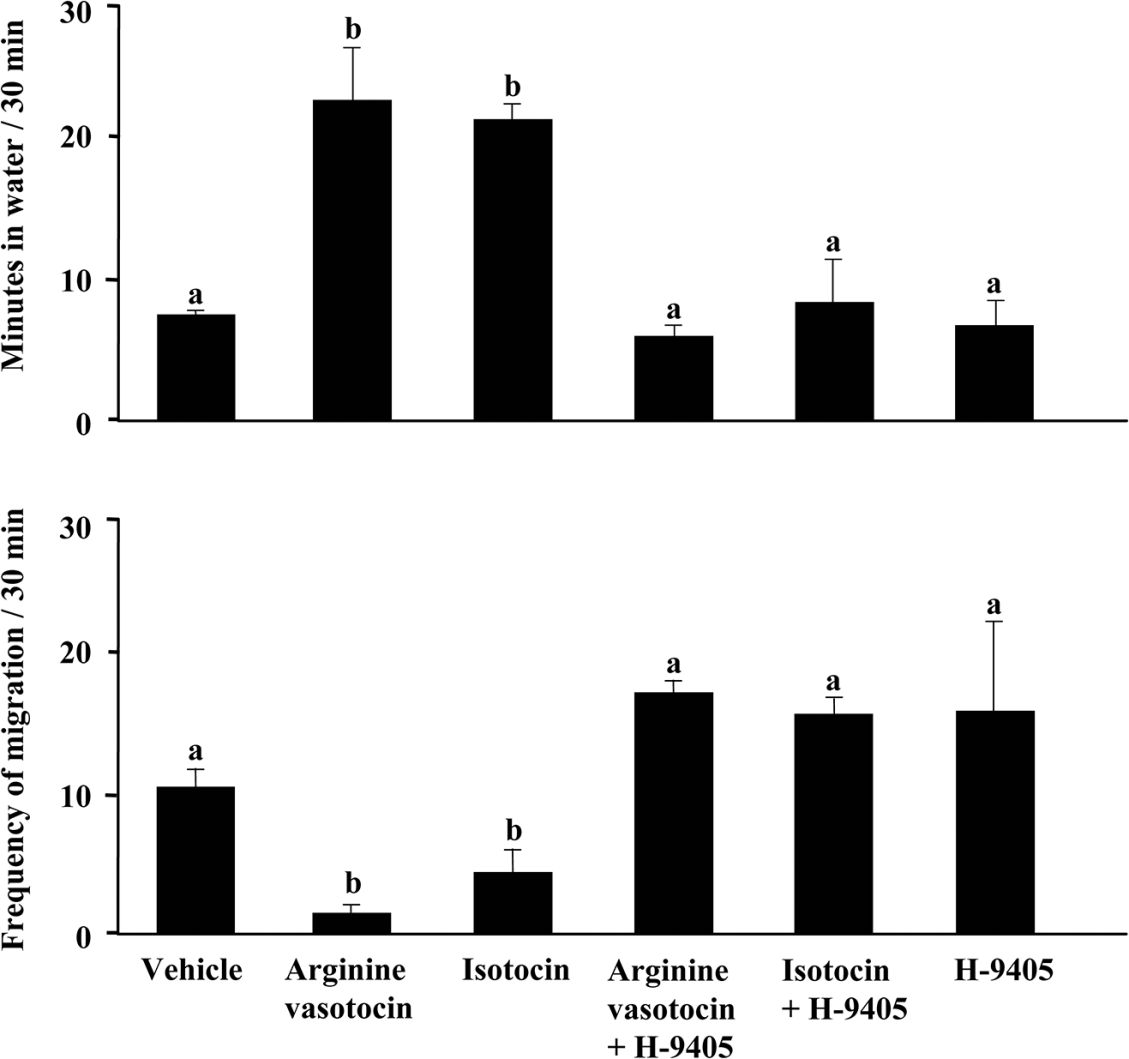
Amphibious behaviour in mudskippers after intracerebroventricular (ICV) injection of a specific isotocin-receptor antagonist. Period of time in water and ‘frequency of migration’ for mudskippers 1 h after ICV injection. Fish were injected with artificial cerebrospinal fluid or 500 pg/g arginine vasotocin or isotocin in the presence of a specific isotocin-receptor antagonist (H-9405) or in the absence of H-9405. Data are shown as the mean ± standard error (SE) of 4 to 9 fish. Only data from ICV procedures whose accuracy was confirmed by Evans blue were used. Different letters above bars indicate a significant difference (P < 0.05).

Mudskippers exhibited a significantly increased period of time in water at 1 h after ICV treatment with high dose VT or IT, compared with control treated with ICV ACSF (Fig 3, left panel). The ‘frequency of migration’ was significantly lower after 1 h of ICV treatment with high dose VT or IT compared with control fish (Fig 3, right panel). In addition, we examined the effects of concurrent ICV treatment with an IT-receptor antagonist, H-9405, which abolished the effects of VT and IT on the period in water and the ‘frequency of migration’ (Fig 4). There was no significant effect in treatment with H-9405 alone and no apparent sex differences in these effects.

### Expression of pro-VT and pro-IT mRNAs during terrestrial adaptation

**Fig 5:**
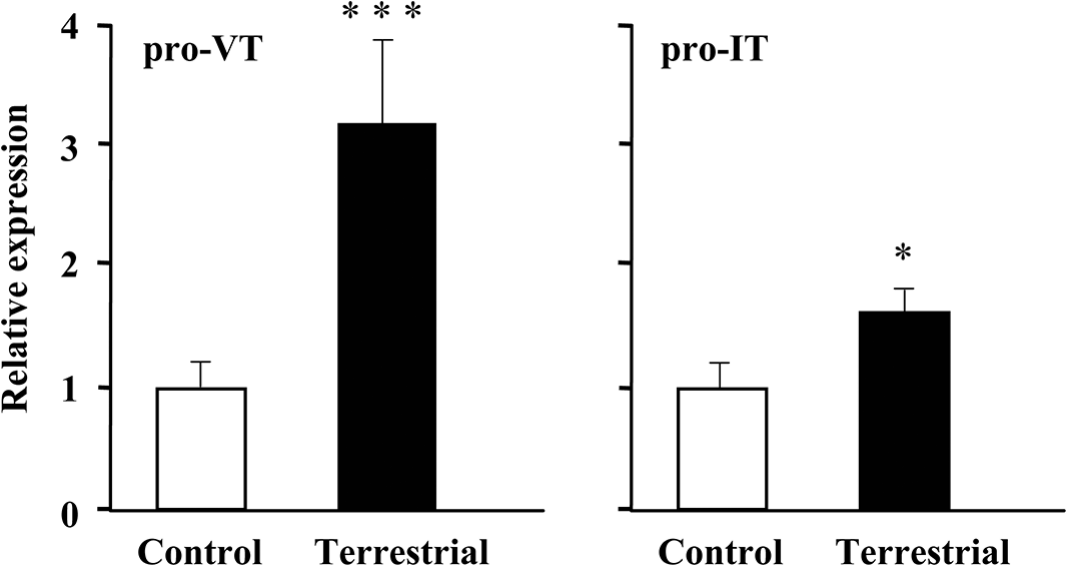
mRNA expression of arginine vasotocin and isotocin precursors in brain of mudskippers under terrestrial conditions. Relative mRNA expression of arginine vasotocin (VT) and isotocin (IT) precursors in brain of mudskippers under terrestrial conditions (5 fish) and controls (10 ppt seawater, 10 fish) for 5 h. Data are shown as the mean ± standard error (SE). *P < 0.05, ***P < 0.001.

Exposure of mudskippers to terrestrial conditions increased brain mRNA levels for pro-VT and pro-IT (Fig 5). The increases in mRNA levels of VT and IT were 3- and 1.5-fold that of controls, respectively, after 5 h. There were no apparent sex differences.

### Localisation and distribution of VT- and IT-immunoreactive neurones

**Fig 6:**
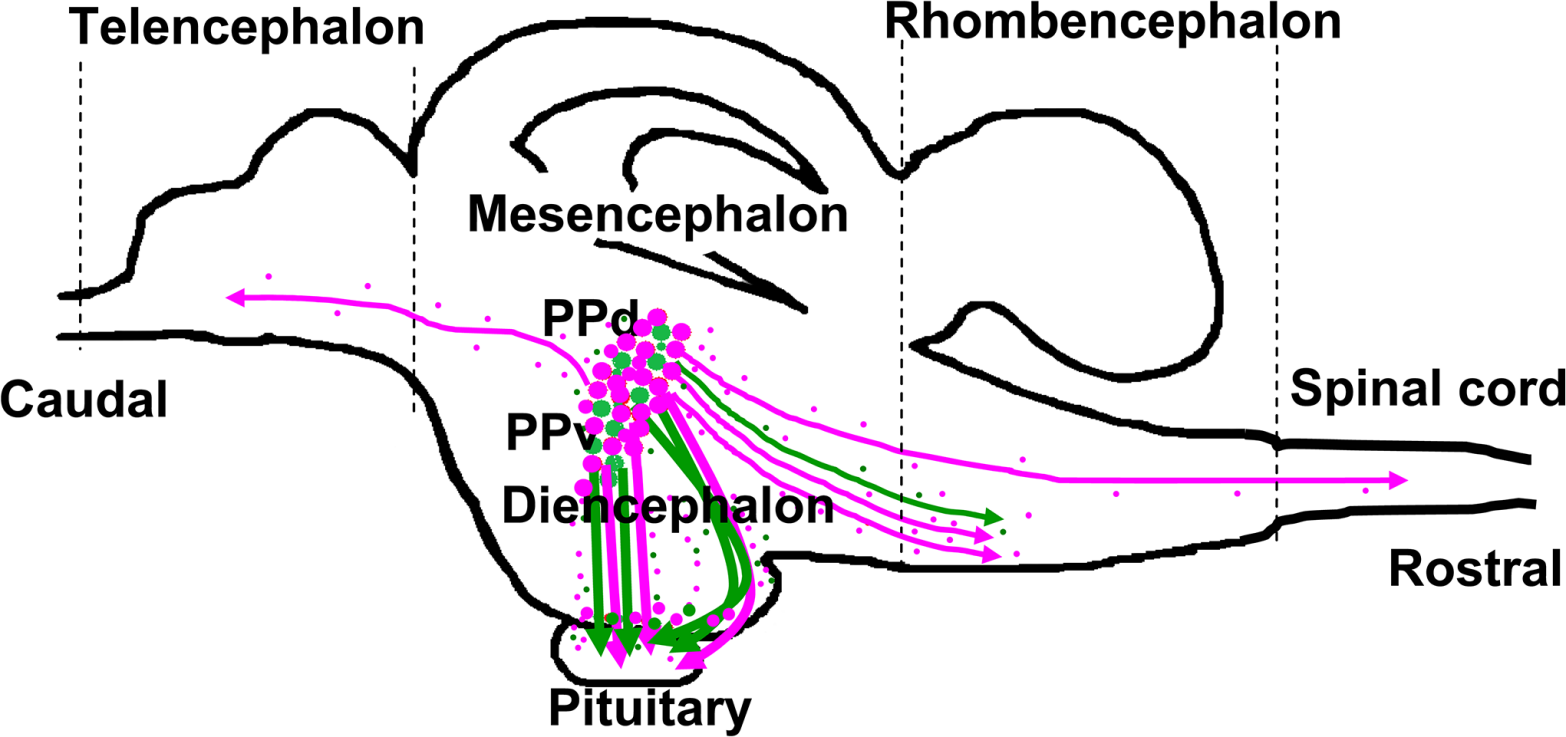
Schematic of arginine-vasotocin (VT)- and isotocin (IT)- immunoreactivities in mudskipper brain. VT- (magenta) and IT- (green) immunoreactive cell bodies and fibres in the brain are shown by solid circles and small dots, respectively. The density of symbols is proportional to the relative density of immunoreactive elements. The *upper part* and *right side* of the schematic drawing show the dorsal side and caudal end of the brain, respectively. Dorsal part of the paraventricular preoptic nucleus (PPd); ventral part of the PP (PPv).

**Fig 7:**
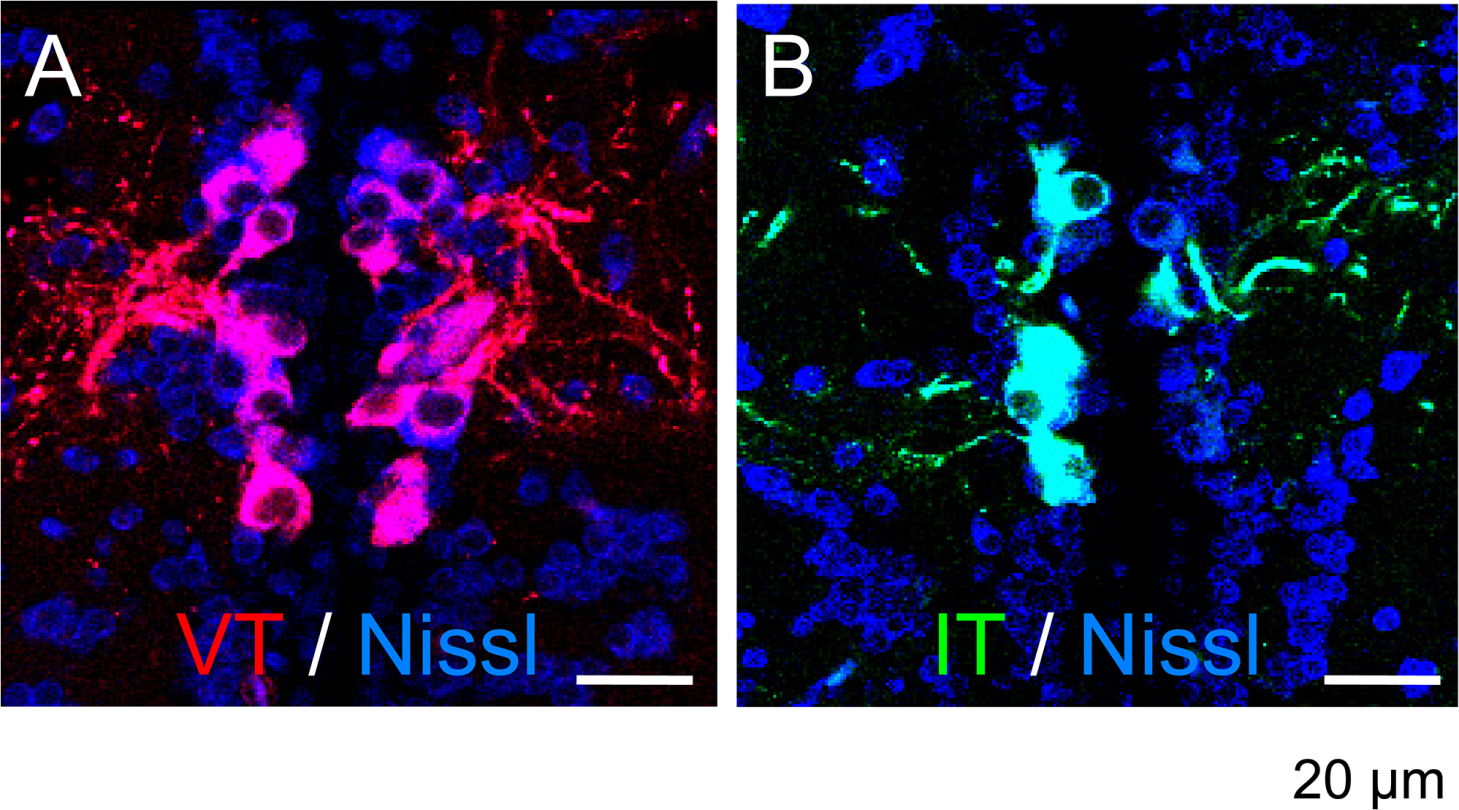
Arginine-vasotocin- (VT) and isotocin- (IT) immunoreactivities in the paraventricular preoptic nucleus (PP) of the hypothalamus. Intense VT- (red) and IT- (green) immunoreactivities were observed in the somata and proximal dendrites of relatively large neurones in transverse sections. Histology of the PP was visualized by blue fluorescent Nissl staining.

**Fig 8:**
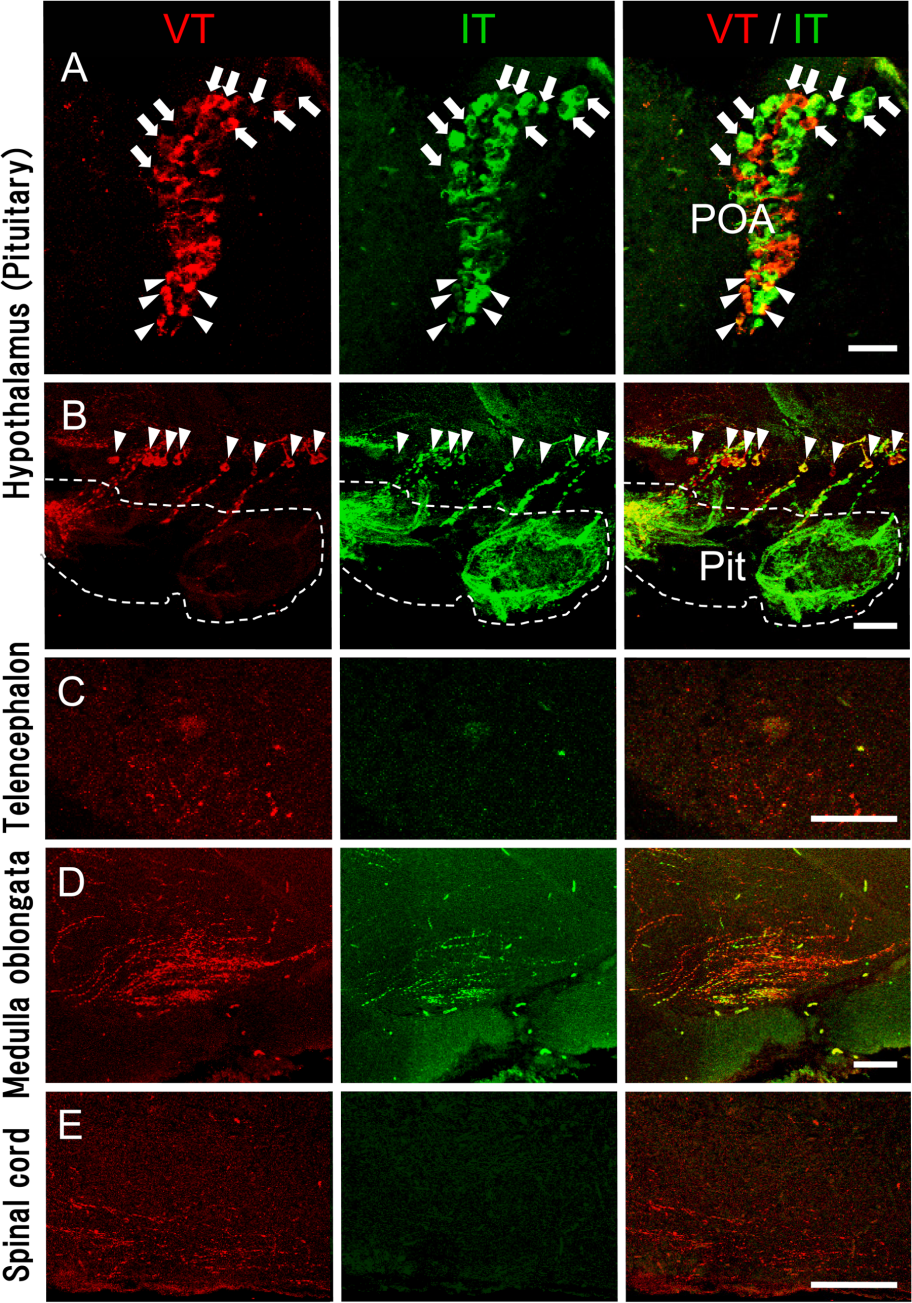
Dual immunofluoresscent analyses in sagittal sections of mudskipper brain. Arginine-vasotocin- (VT) (red) and isotocin- (IT) (green) immunoreactive cell bodies and fibres were found in similar or different populations in several brain regions. Representative photomicrographs are shown for the hypothalamus (A, B), telencephalon (C), medulla oblongata (D), and spinal cord (E). Dorsal is up and caudal is left. (A, B) The paraventricular preoptic nucleus (PP) of the hypothalamus and the ventral hypothalamic area (around the pars tuberalis) contained both VT- and IT-immunoreactive cell bodies. Note that several parvocellular neurones (*e*.*g*., arrowheads) are much smaller than neurones in the magnocellular part of the PP (*e*.*g*., arrows). (C) The telencephalon had relatively sparse VT-immunoreactive fibres, but no IT, in the ventromedial telencephalic area. (D) In the rhombencephalon, a small amount of both VT- and IT-immunoreactive fibres was found in the ventromedial part of the medulla oblongata. (E) A population of VT-immunoreactive fibres, but no IT, was observed in the ventral part of the cervical spinal cord. All displayed sections were cut sagittally. Immunohistochemical studies were repeated independently four times in different fish and gave similar results. Pit, pituitary. Scale bars = 50 μm in A-E.

To assess possible brain regions involved in regulation of amphibious behaviour by neurohypophysial hormones, we examined the localisation and distribution of VT- and IT-immunoreactive cells in the brain, spinal cord, and pituitary of adult mudskippers. As shown schematically (Fig 6), VT- and IT-immunoreactive cell bodies or fibres were found in several telencephalic, diencephalic, mesencephalic, and rhombencephalic regions in the mudskipper brain. In the diencephalon, intense VT (red) and IT (green) immunoreactivities were observed in the somata and proximal dendrites of relatively large (magnocellular) neurones and in those of small sized (parvocellular) neurones in the paraventricular preoptic nucleus (PP) of the hypothalamus (Fig 7). The number of immunoreactive neurones and the density of immunoreactive fibres appeared to be greater for VT (Fig 7A) compared to IT (Fig 7B); the histology of the PP was visualized in blue by fluorescent Nissl staining (Fig 7A and 7B). Dual immunofluorescent analyses in sagittal sections revealed VT- (red) and IT- (green) immunoreactive cell bodies and fibres in several brain regions (Fig 8). The immunoreactive magnocellular neurons were mainly observed in the dorsal part of the PP (PPd), and the parvocellular neurons in the ventral part (PPv). In particular, in the PPd, large somata of VT- and IT-immunoreactive neurones were present in each population (Fig 8A; arrows). Conversely, IT immunoreactivities were seen in some VT-immunoreactive neurons in the PPv (Fig 8A; arrowheads). Small neurones with VT- and IT-immunoreactivities were also found in the ventral hypothalamic area (around the pars tuberalis) (Fig 8B; arrowheads), and the characteristics of these immunoreactive neurones appeared similar to those of the parvocellular part of PP (Fig 8A and 8B; arrowheads). The dense projections of VT- and IT-immunoreactive fibres were present in the ventral part of the hypothalamus and extended into the pars nervosa of the pituitary (Fig 8B). Most of the immunoreactive fibres in the hypothalamus were found at similar distributions for VT and IT and corresponded to projections from the PP, but their fibres somewhat differed in the populations (Fig 8B).

Sagittal sections also revealed that axonal projections were greater for VT than for IT throughout the brain. In the ventromedial telencephalic area, sparse VT-immunoreactive fibres were found, but not IT (Fig 8C). In the rhombencephalon, VT- and IT-immunoreactive fibres were found in the ventromedial part of the medulla oblongata where some VT- and IT-immunoreactive fibres co-localised, but other fibres of a different population expressed only VT (Fig 8D). In contrast to the medulla oblongata, a population of VT-immunoreactive fibres, but not IT, was found in the ventral part of the cervical spinal cord (Fig 8E). There were no apparent sex differences in the localisation or distribution of VT- and IT- immunoreactive fibres.

## Discussion

The mudskipper, an amphibious teleost, preferred the aquatic habitation and tended to stay in water, when treated with VT or IT peripherally or centrally. The discovery that multiple V1a-type, V2-type and IT receptors arose via genome duplications in the evolutionary history of actinopterygian fishes suggests that several VT/IT receptors could be involved in regulation of the amphibious behaviours. Here, stimulation of this behavior by both VT and IT was inhibited by the IT receptor blocker, H-9405. Expression studies of teleost V1a-type receptors (shown to mediate many behaviours [5,36]) and IT receptors in mammalian cell lines indicate that V1a is nearly specific to VT, whereas the sensitivity of the IT receptor to IT is 3-10-times higher than that to VT [37,38,39,40]. Thus, we demonstrated possible involvement of the brain IT receptor in the preference of mudskippers for an aquatic habitat. However, other VT/IT receptors, especially V2-type, may be implicated in the aquatic preference. The V2-type receptor in the hypothalamus and osmoregulatory organs is suggested to play a role in maintaining hydromineral balance [44,45,46,47,48,49]. Two distinct isoforms of IT receptor may also be present in some teleost species [our unpublished results] and full biochemical characterization of the VT/IT receptors is needed.

Brain mRNA levels of pro-VT increased markedly after mudskippers moved to terrestrial conditions, and a moderate increase was seen in pro-IT mRNA levels. Given the relatively wide distribution of VT-positive fibres throughout the brain, increased VT and IT under terrestrial conditions may act at least in part as ligands for brain IT receptors to naturally stimulate the preference of mudskippers for an aquatic environment. The involved nuclei were not identified, but neurons co-expressing VT and IT in the parvocellular part of the paraventricular preoptic nucleus (PP) and in the tuberal nuclei of the hypothalamus may play a role in this behaviour. The distribution of VT/IT fibres provides a foundation for understanding how these peptides modulate behaviour (*i*.*e*., candidate areas for their involvement in regulation of behavior), and this information will be useful for functional manipulation since detailed descriptions of the receptors are almost lacking in teleosts [50,51].

Sensory circumventricular organs (CVOs): the subfornical organ (SFO) and organum vasculosum of the lamina terminalis (OVLT) in the forebrain, and the area postrema in the medulla oblongata, appear to be important for drinking behaviour [52], although the teleost equivalent of mammalian SFO and OVLT have yet to be fully identified [53,54,55]. We have found immunoreactive VT fibres in these brain regions of mudskippers, and IT receptors appear to be present at least throughout the forebrain regions in an African cichlid fish [51], suggesting that, in mudskipper, VT may act on IT receptors that transmit the signal to the neural pathway that stimulates drinking and aquatic preference. In teleosts, however, little is known about the role of IT in influencing the brain to regulate such physiological and behavioural adaptations. In rats, ICV injection of an oxytocin receptor antagonist controls drinking and blood pressure, possibly by stimulating parts of the CVO [56,57].

Regulation of social behaviour in teleosts has been extensively studied in the VT system, rather than IT. Central administration of VT in some species, including the mudskipper, supports a brain-mediated role of VT in modulating aggression, although the directionality (stimulation/inhibition) appears to vary [5,24]. A stimulation study [58] and the presence of V1a [36,50,51] suggested that VT acts through the V1a receptor in the ventromedial telencephalic area and PP to regulate aggression in teleosts. In the mudskipper, we found VT fibers in these regions and showed that pro-VT mRNA levels in the subordinate were higher than in the dominant [24]. This suggests that VT can act via the equivalent V1a in the subordinate to inhibit general aggressive behaviour (i.e. replace, attack, chase, bite), and at least in part via IT receptors to stay in the aquatic habitat forced by the dominant animal.

The findings of this study suggest that the amphibious mudskipper fish is an intriguing model to explore the central role of VT associated with behaviours related to both osmoregulation and aggression. Further analysis is required to determine whether the environmental preference regulated by neurohypophysial hormones is also characteristic of other species. To acquire a clearer picture of the physiological and morphological characteristics associated with these behavioural functions, future studies should focus on a possible role of VT/IT in drinking behaviour, as well as using molecular tools to determine the gene and protein expression patterns of VT/IT receptors in the brain. Recent findings in the fish brain and other important organs/tissues (e.g., gills) suggest that the physiological effects (e.g., social context, hydromineral balance) of a change in VT/IT are dependent on coordinated changes in VT/IT receptor expression, including the localisation, type and abundance of different receptors [36,44,47,59]. Therefore, better integrated assessment of the distribution, function and expressional regulation of these receptors concurrently with evaluation of the hormone production will provide a fuller understanding of how social interactions and environmental factors synergise to influence variation in VT/IT function [60].
